# End-stage renal disease: a risk factor of deep neck infection – a nationwide follow-up study in Taiwan

**DOI:** 10.1186/s12879-017-2531-5

**Published:** 2017-06-13

**Authors:** Geng-He Chang, Ming-Shao Tsai, Chia-Yen Liu, Meng-Hung Lin, Yao-Te Tsai, Cheng-Ming Hsu, Yao-Hsu Yang

**Affiliations:** 1 Department of Otolaryngology, Chang Gung Memorial Hospital, Chiayi, Taiwan; 2 Center of Excellence for Chang Gung Research Datalink, Chang Gung Memorial Hospital, Chiayi, Taiwan; 3 Graduate Institute of Clinical Medical Sciences, College of Medicine, Chang Gung University, Taoyuan, Taiwan; 4 Institute of Occupational Medicine and Industrial Hygiene, National Taiwan University College of Public Health, Taipei, Taiwan; 5 Department of Traditional Chinese Medicine, Chang Gung Memorial Hospital, Chiayi, Taiwan; 6 School of Traditional Chinese Medicine, College of Medicine, Chang Gung University, Taoyuan, Taiwan; 7 No.6, W. Sec., Jiapu Rd., Puzih City, Chiayi County 613 Taiwan

**Keywords:** Abscess, Cellulitis, Cervical, Dialysis, ESRD, Failure, Kidney, Nephropathy, NHIRD, Predisposing

## Abstract

**Background:**

Uremia is likely a risk factor for deep neck infection (DNI). However, only a few relevant cases have been reported, and evidence sufficient to support this hypothesis is lacking. The aim of the study is to investigate the effects of end-stage renal disease (ESRD) on DNI.

**Methods:**

We used the database of the Registry for Catastrophic Illness Patients (RFCIP), a subset of the National Health Insurance Research Database (NHIRD) in Taiwan, to conduct a retrospective follow-up study. Between 1997 and 2013, a total of 157,340 patients in Taiwan with ESRD who received dialysis were registered in the RFCIP, whom were matched with a database consisting of 1,000,000 randomly selected patients who represented the national population, to conduct the follow-up study for investigating the incidence of DNI in the ESRD and control cohorts.

**Results:**

In the ESRD group, 280 DNIs were identified with an incidence rate of 43 per 100,000 person-years. In the comparison group, 194 DNIs were identified with an incidence rate of 20 per 100,000 person-years. The incidence rate ratio was 2.16 (*p* < 0.001). Kaplan–Meier analysis indicated that the ESRD group had a significantly higher cumulative incidence of DNI (*p* < 0.001). According to Cox regression analysis, the hazard ratio of ESRD for DNI was 2.23 (*p* < 0.001). The therapeutic methods (non-surgery and surgery), performance of tracheostomy, duration of hospitalization did not differ significantly between the two groups, except more ESRD-DNI patients were admitted to intensive care units. The mortality rate of patients with DNI in the ESRD group was significantly higher than that in the control group (8.6% for ESRD vs 3.6% for control, *p* = 0.032). Furthermore, the Kaplan–Meier analysis demonstrated a poorer survival outcome in the ESRD group (*p* = 0.029). However, the individual survival outcomes following non-surgical and surgical therapies in the ESRD group did not differ significantly (*p = 0.31*).

**Conclusions:**

ESRD is a predisposing factor for DNI, increasing its risk by twofold. In the patients with ESRD, DNI was not associated with higher rates of surgical debridement, tracheostomy, and mediastinal complications or longer hospital stays; however, it was associated with poorer survival outcomes, regardless of the therapeutic method.

## Background

Deep neck infection (DNI) is a common disease and usually presents at emergency departments, which usually requires intensive care and aggressive treatment. The wide availability of antibiotics, improvement of diagnostic technology, and the concept of early surgical debridement had significantly reduced the morbidity and mortality of DNI [1, 2]. However, DNI remains a potentially life-threatening disease to date, particularly among elderly individuals and patients with systemic diseases [3–5]. Diabetes mellitus (DM) has been considered a definite predisposing systemic disease in 17%–34% of DNIs [4–8]. Other systemic diseases, such as uremia, liver cirrhosis, and autoimmune disease, are also considered risk factors for DNI [4, 9, 10].

Wang (2003) analyzed 196 DNI cases retrospectively and considered uremia a risk factor for complicated DNI [9]. Huang (2004) published a case series involving of 185 patients with DNI and found that patients with systemic diseases were predisposed to more severe DNI and more complications than were individuals without systemic diseases [4]. In Huang’s study, a total of six patients with uremia were included in the systemic disease group. However, the two aforementioned studies provided no information about the severity of uremia and conducted no further investigation on the influence of uremia on DNI. Moreover, we reviewed relevant studies conducted worldwide, but no case of DNI with end-stage renal disease (ESRD) has been reported [3, 5–8, 11–19].

ESRD is widely prevalent, particularly in Taiwan, which has the highest global incidence rate for renal replacement therapy [20–22]. Patients with ESRD are potentially immunocompromised and predisposed to infection with higher morbidities and mortality than those without ESRD [23–25]. Therefore, research regarding the effect of DNI on patients with ESRD is crucial and warrants more attention.

To our knowledge, no studies investigating the effects of ESRD on DNI have been published. Hence, we used the National Health Insurance Research Database (NHIRD) in Taiwan to conduct a retrospective cohort study to investigate these effects, particularly concerning the incidence rate and risk of DNI (primary endpoints), as well as its interventions, complications, and survival outcomes (secondary endpoints).

## Methods

### Database—National Health Insurance Research Database

In March 1995, the Taiwan government started the National Health Insurance Program and accordingly enacted an insurance policy, which considerably increased the national insurance coverage from 60% to 92%. By 2016, the coverage reached 99.6% [26]. In 2000, the Taiwan government established the National Health Insurance Research Database (NHIRD), which comprises data on all insurants, and these data are processed to being deidentified and released for research [27]. With certificate of ethical approval, the data can be accessed by way of clinician/institute application to the Bureau of National Health Insurance. The NHIRD provides all data generated during the reimbursement for insurance, including diagnosis sets for outpatients and inpatients, prescription drugs and doses, examinations, procedures, surgeries, payments, resident locations, and income levels. The diagnosis sets consist of three codes for outpatient visits and five codes for hospitalization. These codes are based on the International Classification of Diseases, Ninth Revision, Clinical Modification (ICD-9-CM).

We conducted the study in accordance with the guidelines of the Declaration of Helsinki. This study was exempted from obtaining informed consent from the participants, because the database was nationwide and the data were deidentified. All information of the insurants was unidentifiable, and this study did not violate their rights or adversely affect their welfare. The research was approved by the Institutional Review Board of Chang Gung Memorial Hospital (IRB number: 201601249B1).

### Study group—Registry for catastrophic illness patients

In the Taiwan health insurance system, the category of catastrophic illness patients (CIP) has been instituted for a particular group who benefit from the treatment of relevant catastrophic illnesses. The verification of the eligibility for CIP certification requires strict investigation. For instance, a patient who has relevant diagnosis codes for ESRD and has received renal replacement therapy for at least 3 months can apply for CIP certification. Patients with CIP certification for ESRD pay concessional fees for ESRD-associated therapy, including dialysis. Insurants with CIP certification are categorized in the Registry for Catastrophic Illness Patients (RFCIP) associated with the NHIRD.

Using the RFCIP, we defined a study cohort consisting of patients with ESRD who received dialysis from 1 January 1997 to 31 December 2013 in Taiwan. We used the following ESRD-associated ICD-9 codes, which was defined for RFCIP [27]: 585 (chronic renal failure), 586 (unspecific renal failure), 403.01 (malignant hypertensive renal disease with renal failure), 403.11 (benign hypertensive renal disease with renal failure), 403.91 (unspecified hypertensive renal disease with renal failure), 404.02 (malignant hypertensive heart and renal disease with renal failure), 404.03 (malignant hypertensive heart and renal disease with congestive heart failure and renal failure), 404.12 (benign hypertensive heart and renal disease with renal failure), 404.13 (benign hypertensive heart and renal disease with congestive heart failure and renal failure), 404.92 (unspecified hypertensive heart and renal disease with renal failure), and 404.93 (unspecified hypertensive heart and renal disease with congestive heart failure and renal failure). In total, 157,340 patients were included in the study cohort.

### Control group—LHID2000 database

For providing adequate data for research, until 2016, the NHIRD published three databases composed of 1,000,000 insurants representing all insurants in Taiwan, and these databases were named Longitudinal Health Insurance Database 2000 (LHID2000), LHID2005, and LHID2010. For example, LHID2000 consists of 1,000,000 insurants in 2000 who were randomly statistically selected (Oracle’s internal random number generator) from all insurants in Taiwan. No statistically significant differences exist in age, sex, or health care costs between the LHID2000 sample group and all enrollees, according to a National Health Research Institutes report [27], and LHID2000 has been used in several population-based studies [28–30]. Therefore, we used LHID2000 to generate a control cohort that was matched with the study cohort according to sex, age, urbanization level, income level, DM, and hypertension and analyzed the effects of DNI on the two cohorts.

### Main outcome – Incidence of DNI

A DNI was defined as a severe infection in the deep neck that required hospitalization for intensive care. Therefore, while determining the incidence of DNI in the study and control cohorts, we searched for diagnoses during hospitalization that included the following ICD-9 codes: 528.3 (cellulitis and abscess of oral soft tissues; Ludwig angina), 478.22 (parapharyngeal abscess), 478.24 (retropharyngeal abscess), and 682.11 (cellulitis and abscess of neck) [28]. We excluded patients with a previous inpatient diagnosis of DNI before ESRD dialysis to ensure the validity of results concerning the predisposition of ESRD for DNI; consequently, 503 patients were excluded from the study group. After exclusion, 156,837 individuals with ESRD were enrolled in the study group.

### Matching process

**Fig. 1:**
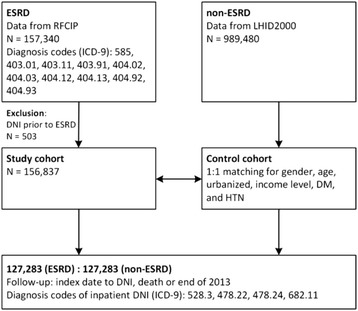
Enrolment schema of the study and comparison cohorts. Patients with ESRD were identified since 1997 to 2013 in Taiwan from RFCIP database, and a total of 157,340 cases were collected. 503 cases were excluded due to DNI occurrence prior to the index date of ESRD. A total of 156,837 cases with ESRD were eligible for a study cohort. LHID2000 database consisting of 989,480 insurants representing the general population in Taiwan was used to match with the study cohort for gender, age, urbanized, income level, DM and HTN with 1:1 fusion. Finally, there were 127,283 patients with ESRD (study group) and 127,283 patients without ESRD (control group) to conduct the follow-up study. The follow-up ended on DNI identified based on the ICD-9 codes of 528.3, 478.22, 478.24, and 682.11, death, or the end of 2013. Abbreviations: ESRD, end-stage renal disease; RFCIP, Registry for Catastrophic Illness Patients; LHID2000, Longitudinal Health Insurance Database 2000; DNI, deep neck infection; ICD-9, International Classification of Diseases, Ninth Revision; DM, diabetes mellitus; HTN, hypertension

For each ESRD case, one control without ESRD (1:1) with matching gender, age, urbanization level, income level, DM and hypertension was randomly selected from the LHID2000 database to form a control group. The index date of the study group was the date of registry in the RFCIP for patients with ESRD, and an index date matching that of patients with ESRD was created for the control group. DNI diagnosed before the index date was excluded. A total of 127,283 controls matching the study patients were included. DNI diagnosis codes were used in the two groups to determine the incidence rate of DNI with a follow-up to the end of 2013. The follow-up period was from the index date to the diagnosis of DNI, and some patients were censored because of death (Fig. 1).

### Comorbidities

The following comorbidities were defined using ICD-9-CM codes recorded in the claims data: DM (ICD-9-CM code: 250), hypertension (ICD-9-CM codes: 401–405), liver cirrhosis (CD-9-CM codes: 571.2, 571.5–571.6), systemic autoimmune disease (ICD-9-CM codes: 443.1, 446.0, 446.2, 446.4–446.5, 446.7, 696.0–696.1, 710.0–710.4, 714.0–714.4), chronic obstructive pulmonary disease (COPD) (ICD-9-CM codes: 491,492,496), coronary artery disease (CAD) (ICD-9-CM codes: 410–414), and cerebrovascular accident (CVA) (ICD-9-CM codes: 430–438) [28, 31, 32]. Medical comorbidities were included if they appeared one or more times in the diagnoses of inpatients or three or more times in the diagnoses of outpatients. Comorbidities were included if they occurred within 12 months before the index date.

### Therapy classification

We assessed the therapeutic methods used for treating patients with DNIs in the two groups. The treatment methods were divided into three subgroups: antibiotics alone, antibiotics with abscess aspiration or drainage, and surgical intervention. The intervention, including abscess aspiration or drainage and surgical debridement, was identified using the claims records during each hospitalization for DNI treatment. If the patients received surgical intervention, they were included in the surgery subgroup, irrespective of the performance of abscess aspiration or drainage. If the patients received abscess aspiration or drainage without surgery, they were included in the aspiration subgroup. If the patients received only antibiotic treatment during hospitalization, they were included in the antibiotic subgroup.

### Prognosis evaluation

For evaluating prognosis, we analyzed the duration of hospitalization, care in intensive care units (ICUs), performance of tracheostomy, and mediastinal complications, which were defined according to the receipt of mediastinal surgery during hospitalization or the following ICD-9-CM codes: 510, 513 and 519.2. The mortality and mediastinitis-related mortality were also investigated in both cohorts. Mortality was defined as death occurring during DNI treatment. The mediastinitis-related mortality was defined as death during DNI treatment accompanied by a diagnosis of mediastinitis.

### Statistical analysis

The sociodemographics and incidence rate of DNI were compared between the ESRD and control groups using the Pearson’s chi-squared test. The Fisher exact test was used to compare the rates of tracheostomy, mediastinitis, and mortality. The duration of hospitalization was compared using the Student’s t test. The results of Kaplan–Meier analysis revealed the cumulative incidence of DNI in both groups. Furthermore, the analysis was used to investigate the survival outcomes. The risk of DNI for the study group was estimated using a Cox proportional hazard model. All the analyses were performed using SAS software, version 9.4 (SAS Institute, Cary, NC), and the level of statistical significance was set at *p* < 0.05.

## Results

Table 1 illustrates the distribution of sociodemographic characteristics as well as the DNIs and comorbidities identified in the study and comparison cohorts after the matching process. The ESRD cohort had a significantly higher prevalence of liver cirrhosis, systemic autoimmune disease, CAD, and CVA, but not COPD than the non-ESRD cohort (Table 1). In the 127,283 ESRD patients, 280 (0.2%) DNIs were identified. The incidence rate was 43 per 100,000 person-years in a mean follow-up period of 5.12 ± 4.48 years. By contrast, among the 127,283 non-ESRD controls, 194 DNIs (0.1%) were identified in a mean observation period of 7.67 ± 4.65 years with an incidence rate of 20 per 100,000 person-years. The incidence rate ratio was 2.16 with a 95% confidence interval (CI) of 1.8 to 2.6. The incidence of DNI was significantly higher in the ESRD cohort than the control one (*p* < 0.001).

**Table 1:** Demographic and characteristics between the ESRD and Non-ESRD groups

Characteristic	ESRD		Non-ESRD		*p*-value^a^
	*N*	%	*N*	%	
Total	127,283		127,283		
Gender					1
Male	64,306	50.5	64,306	50.5	
Female	72,902	49.5	72,902	49.5	
Age (yrs)					1
< 65	68,698	54.0	68,698	54.0	
≥ 65	58,585	46.0	58,585	46.0	
Urbanized level					1
1 (City)	35,299	27.7	35,299	27.7	
2	57,162	44.9	57,162	44.9	
3	22,092	17.4	22,092	17.4	
4 (Village)	12,730	10.0	12,730	10.0	
Income (NTD, per month)					1
0	26,695	21.0	26,695	21.0	
1–15,840	20,261	15.9	20,261	15.9	
15,841–25,000	60,617	47.6	60,617	47.6	
≥ 25,001	19,710	15.5	19,710	15.5	
DNI	280	0.2	194	0.1	<0.001
Comorbidities					
DM	71,155	55.9	71,155	55.9	1
HTN	117,545	92.4	117,545	92.4	1
Autoimmune	8345	6.6	7406	5.8	<0.001
Liver cirrhosis	12,559	9.9	5588	4.4	<0.001
CAD	69,109	54.3	48,835	38.4	<0.001
CVA	49,042	38.5	38,602	30.3	<0.001
COPD	30,454	23.9	30,704	24.1	0.246

*Abbreviations*: *DNI* deep neck infection, *ESRD* end-stage renal disease, *NTD* New Taiwan dollar, *DM* diabetes mellitus, *HTN* hypertension, *CAD* coronary artery disease, *CVA* cerebrovascular accident, *COPD* chronic obstructive pulmonary disease

^a^Pearson’s chi-squared tests

Table 1 revealed the demographic and clinical characteristics between the ESRD and Non-ESRD groups. Gender, age, urbanized, income level, DM and HTN were balanced between the two groups. The incidence of DNI was significantly higher in the ESRD group (*p* < 0.001)

**Fig. 2:**
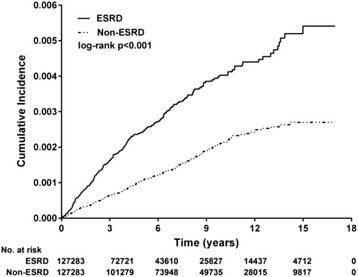
Cumulative incidence of DNI for ESRD versus non-ESRD. The Kaplan-Meier analysis demonstrated the cumulative DNI identified in the study and control cohorts, respectively, during the follow-up period (1997–2013). The log-rank test revealed a significantly higher cumulative incidence in the ESRD group (*p* < 0.001)

The Kaplan–Meier analysis indicated the cumulative incidence of DNI in both cohorts in the observation period from 1997 to 2013. Log-rank analysis indicated that the ESRD group had a significantly higher incidence of DNI (*p* < 0.001) (Fig. 2). The Cox proportional hazard model was employed to analyze the crude and adjusted hazard ratios (HRs) for both groups after adjustment for gender, age, urbanization level, income level, and the selected comorbidities. The risk of DNI in the patients with ESRD was 2.23-fold greater than that in the patients without ESRD (HR for DNI in ESRD group: 2.23, 95% CI: 1.84–2.69, *p* < 0.001) (Table 2). In the Cox regression analysis, DM, hypertension, and a lower urbanization level were significant risk factors for DNI; however, the effects of liver cirrhosis, systemic autoimmune disease, COPD, and CVA on DNI were not statistically significant. In addition, patients with CAD had a lower risk of DNI.

**Table 2:** Multivariable Cox proportional hazards regression of the association between DNI and potential risk factors

Variables	Crude	95% CI	*p*-value	Adjusted	95% CI	*p*-value
	HR			HR		
ESRD vs Non-ESRD						
Non-ESRD	1.00			1.00		
ESRD	2.09	(1.74–2.51)	<0.001	2.23	(1.84–2.69)	<0.001
Gender						
Male	1.00			1.00		
Female	0.97	(0.81–1.17)	0.766	0.93	(0.77–1.12)	0.453
Age						
< 65	1.00			1.00		
≥ 65	1.04	(0.87–1.24)	0.692	1.12	(0.92–1.36)	0.283
Urbanized level						
1 (City)	1.00			1.00		
2	1.03	(0.82–1.29)	0.799	1.03	(0.82–1.29)	0.829
3	1.22	(0.93–1.60)	0.155	1.23	(0.93–1.62)	0.145
4 (Village)	1.37	(1.01–1.87)	0.046	1.40	(1.02–1.93)	0.039
Income (NTD, per month)						
0	1.00			1.00		
1–15,840	1.23	(0.92–1.64)	0.167	1.20	(0.89–1.61)	0.240
15,841–25,000	1.06	(0.83–1.34)	0.645	0.96	(0.75–1.24)	0.773
≥ 25,001	0.82	(0.59–1.14)	0.242	0.76	(0.54–1.08)	0.125
Comorbidities						
DM	1.41	(1.17–1.70)	<0.001	1.50	(1.24–1.82)	<0.001
HTN	1.57	(1.07–2.31)	0.020	1.57	(1.06–2.31)	0.025
Autoimmune	1.06	(0.74–1.53)	0.750	1.07	(0.74–1.54)	0.732
Liver cirrhosis	1.22	(0.87–1.71)	0.255	1.01	(0.72–1.43)	0.945
CAD	0.97	(0.81–1.16)	0.751	0.79	(0.65–0.95)	0.014
CVA	1.04	(0.86–1.26)	0.659	0.93	(0.76–1.13)	0.457
COPD	0.90	(0.72–1.12)	0.323	0.88	(0.70–1.11)	0.291

*Abbreviations*: *CI* confidence interval, *HR* hazard ratio

Table 2 demonstrated the Cox regression mode investigating the risk factors for DNI after adjusting the multivariable. After adjustment, the ESRD had 2.23-fold risk for DNI (*p* < 0.001)

**Fig. 3:**
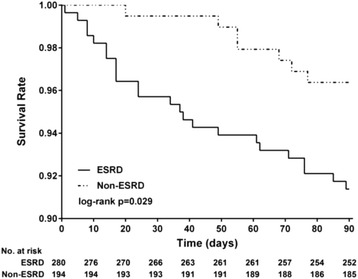
Kaplan-Meier-estimated overall survival distributions for ESRD-DNI versus non-ESRD-DNI. The Kaplan-Meier analysis demonstrated the survival outcomes of the study and control cohorts, respectively, in the 3 months after DNI. The log-rank test revealed a significantly poorer survival result in the ESRD group (*p* = 0.029)

Table 3 presents the therapeutic methods used for treating DNI in both groups, namely antibiotic, aspiration and surgery, and other factors, including the performance of tracheostomy, duration of hospitalization, care in ICUs, mediastinal complications and mortality (Table 3). The proportion of treatment did not differ significantly between the two groups (*p* = 0.394), and proportion of the three therapeutic methods in the ESRD group resembled those in the control group. Nearly three-quarters of patients with DNI were treated using antibiotics (ESRD: 74.3% and control: 75.3%), and one-quarter of the patients received surgical intervention (ESRD: 24.3% and control: 21.7%). Abscess aspiration for DNI treatment accounted for a small portion in both groups (ESRD: 1.4% and control: 3.1%). The performance of tracheostomy (ESRD: 3.6%, control: 3.6%, *p* = 0.983) and duration of hospitalization (ESRD: 11.1 ± 14.7 days, control: 9.7 ± 12.7 days, *p* = 0.269) did not differ significantly between the two groups; however, more patients in the ESRD group received care in ICUs (ESRD: 13.9%, control: 7.2%, *p* = 0.023). In total, seven and two patients developed mediastinal complications in the ESRD and control groups, respectively (ESRD: 2.5%, control: 1%, *p* = 0.249), and approximately half of those DNI cases with mediastinal complication result in mortality (ESRD: 3/7, 43%, control: 1/2, 50%). The duration of hospitalization ranged from 1 to 89 days. In the 3 months after DNI, the patients with ESRD had a significantly higher mortality rate than did the controls (ESRD: 8.6%, control: 3.6%, *p* = 0.032). Kaplan–Meier analysis was used to investigate the survival outcomes, and log-rank tests revealed significantly poorer results in the ESRD cohort during the 3 months after hospitalization for treatment of DNI (*p* = 0.029) (Fig. 3).

**Table 3:** Analysis of therapeutic interventions, complications and prognostic outcomes in patients with DNI

Characteristic	ESRD-DNI		Non-ESRD-DNI		*p*-value
	*N*	%	*N*	%	
Total	280		194		
Therapy					0.394^b^
antibiotic	208	74.3	146	75.3	
aspiration	4	1.4	6	3.1	
surgery	68	24.3	42	21.7	
Tracheostomy	10	3.6	7	3.6	0.983^c^
Hospitalization (mean ± SD)	11.1 ± 14.7		9.7 ± 12.7		0.269^d^
ICU care	39	13.9	14	7.2	0.023^c^
Mediastinitis	7	2.5	2	1.0	0.249^c^
Mediastinitis-Mortality	3	1.1	1	0.5	0.515^c^
Mortality^a^	26	8.0	6	2.9	0.021^c^

^a^Mortality occurrence after DNI

^b^Pearson’s chi-squared tests

^c^Fisher exact tests

^d^Student’s t tests

*Abbreviations*: *SD* standard deviation, *ICU* intensive care unit

Table 3 demonstrated the analysis of three therapeutic methods used for treatment of DNI, the indicators used for evaluating the interventions, complications and prognostic outcomes in both cohorts. There was no difference in the methods for treatment and the proportion of three therapies resembled in both cohorts. Tracheostomy rate, duration of hospitalization and mediastinal complication did not differ in both cohorts; however, the proportion of patients receiving ICU care was higher among those with ESRD and DNI. ESRD group had significantly higher mortality rate than non-ESRD group (*p* = 0.032)

## Discussion

Uremia has been considered a risk factor for DNI [4, 9], but evidence sufficient to support this hypothesis is lacking. The nationwide follow-up study provided a large number of cases for studying the effect of ESRD on DNI and confirmed that it is a predisposing factor associated with a twofold risk of DNI.

In general, the treatment of DNI depends on disease manifestations, airway conditions, laboratory results, and radiological studies. The therapeutic choices are antibiotics alone, antibiotics in combination with abscess aspiration, and surgical debridement. Usually, physicians do not undertake an advanced intervention considering the patients’ underlying disease. A DNI requiring surgical intervention is considered a more serious condition.

According to previous studies, surgical debridement for treating DNI comprises 55%–80% of all therapies [3, 4, 6, 10–13]. Tracheostomy has been reported to be performed in 3.5%–10% of DNIs [3, 5, 9, 11, 12, 33], and Huang stated that the rate was as high as 20% in DNIs with systemic disease [4]. DNIs in patients with medical comorbidities have been reported to cause a longer hospitalization duration and more complications [4, 15]. In our study, the three therapeutic methods accounted for similar proportions of treatments in the ESRD and control groups. Specifically, the proportion of surgical intervention was not higher in the ESRD group than in the control group. Furthermore, the performance of tracheostomy (3.6% in both groups) and duration of hospitalization did not differ significantly between the two groups (Table 3). According to these outcomes, ESRD is a risk factor for DNI; however, it does not result in intervention more advanced than that in patients without ESRD.

Nevertheless, regardless of the presence of ESRD in our study, surgical therapy accounted for a quarter of all therapies (ESRD: 24.3% and control: 21.7%), whereas conservative treatment constituted the remainder. These results are inconsistent with those of previous studies. This inconsistency is likely due to previous studies being conducted in medical centers or tertiary hospitals, which receive and treat more severe DNI cases [3, 4, 6, 10–13]. Hence, surgical intervention was commonly recommended for those cases of severe DNIs. However, we collected the cases in our nationwide study from hospitals across Taiwan. These DNI cases were distributed from primary to tertiary hospitals, including patients with relatively low severity, and thus, our study could provide an overview of DNI treatment and prognosis.

In previous studies, the duration of hospitalization for treating DNI ranged from 2 to 90 days [18, 34], and in our study, the hospitalization duration was 1 to 89 days. We investigated the 3-month mortality in both groups and observed that the patients with ESRD had a higher rate of mortality after DNI (ESRD: 8.6%, control: 3.6%, *p* = 0.032) (Table 3). The Kaplan–Meier analysis also indicated a significantly poorer cumulative survival outcome for the ESRD group in the 3 months after DNI (*p* = 0.029) (Fig. 3). In previous studies, the DNI-related mortality rate was 1%–2.5% [1, 4, 9–12, 18], and the mortality rate in patients with comorbidities or old age tended to be higher (1.5%–5%) [4, 5, 18]. The mortality rate of the control group in our study was in agreement with the preceding data, and the rate in the patients with ESRD and DNI was higher than the previously reported rates. Infection, especially in the lungs, joints, and soft tissue, has been reported to be responsible for the higher morbidity and mortality in patients with ESRD than in those without ESRD, and our findings are consistent with these reports [23–25].

**Fig. 4:**
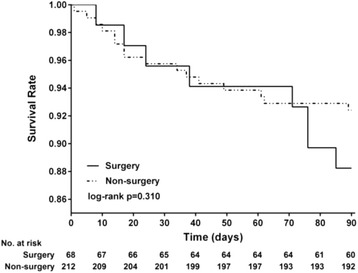
Kaplan-Meier-estimated individual survival distributions from ESRD-DNI patients for surgery versus non-surgery. The Kaplan-Meier analysis demonstrated the individual survival outcomes of “surgery” and “non-surgery” therapies in the ESRD cohort in the 3 months after DNI, and the log-rank test revealed no significant difference between the two therapeutic methods for the survival results (*p* = 0.310)

To investigate the association between therapeutic methods and survival, we classified the therapies for DNI as surgical and nonsurgical. The surgical group consisted of patients who received surgical interventions, which are mainly performed under general anesthesia in an operating room. The nonsurgical group comprised patients treated with antibiotics alone or antibiotics in combination with aspiration, which is usually performed under local anesthesia but not in an operating room. Kaplan–Meier analysis was conducted to determine the individual survival results of the two therapeutic methods (nonsurgery vs surgery) in the ESRD group, and log-rank tests revealed no significant difference in the two methods (*p* = 0.31) (Fig. 4). This result implied that the methods of treatment would be not responsible for the higher mortality. We analyzed the inpatient diagnostic codes for those deaths and noted some medical complications encountered after DNI that may be related to the higher mortality, including CVA, pneumonia, gastrointestinal perforation or hemorrhage, peritonitis, and septic shock. Therefore, we supposed that the higher mortality in ESRD patients after DNI would be related to unanticipated lethal medical sequelae developing after DNI; however, further research should be conducted to investigate the exact causes.

In previous case-series-based studies, the rate of mediastinitis developing after DNI was reported to be 1.7%–2.7% [4, 9, 18, 19], and in reviewing those articles, we observed a high proportion of comorbidities, such as DM, in the DNI cases with mediastinitis. In our study, the patients with ESRD did not have a significantly higher rate of mediastinal complications after DNI in comparison with the controls (ESRD: 2.5%, control: 1%, *p* = 0.249), and the rates of mediastinitis development in both cohorts were relatively consistent with the preceding data. Furthermore, in the aforementioned studies, the mortality rate in patients with DNI and mediastinitis ranged from 40% to 41% [4, 18]. The mediastinitis-related mortality after DNI did not differ significantly between the ESRD and non-ESRD patients in the current study (ESRD: 3/7, 42%; non-ESRD: 1/2, 50%, *p* = 0.515), and the ratio was in accordance with the preceding data. Thus, our findings indicate that ESRD in patients with DNI is not associated with an increased risk of mediastinal complications; nevertheless, these complications entail a high risk of mortality.

Our nationwide study had some limitations. We used ICD-9-CM codes to define patients with DNIs and identified the associated interventions, complications, and mortality in both cohorts to evaluate the prognostic outcomes; however, we could not obtain information concerning the extent of disease, pathogens, laboratory tests, and imaging findings, which is usually used for evaluating the disease severity. The effects of these omitted factors on ESRD and DNI require further investigation. Furthermore, we investigated the 3-month mortality as an alternative to the DNI-specific mortality because the definite cause of death could not be identified using the database; consequently, further research is necessary.

## Conclusions

This nationwide population-based study was the first to investigate the epidemiological data of DNI development in patients with ESRD and the relevant prognosis. It corroborated that ESRD is a predisposing factor for DNI. DNI in patients with ESRD is not associated with higher rates of surgical intervention, tracheostomy, mediastinitis, and mediastinitis-related mortality or a longer duration of hospitalization relative to those of patients without ESRD; however, it would lead to a poorer survival outcome. Such outcomes are not related to the therapeutic intervention, and their cause requires further research.

## Abbreviations

CI: Confidence interval
CIP: Catastrophic illness patients
DM: Diabetes mellitus
DNI: Deep neck infection
ESRD: End-stage renal disease
HR: Hazard ratio
ICD-9-CM: International Classification of Diseases, Ninth Revision, Clinical Modification
ICU: Intensive care unit
LHID2000: Longitudinal Health Insurance Database 2000
NHIRD: National Health Insurance Research Database
RFCIP: Registry for Catastrophic Illness Patients
